# Retraction Note: Demystifying anxiety and demotivation in on-line assessment: a focus on the impacts on academic buoyancy and autonomy

**DOI:** 10.1186/s40359-025-02867-x

**Published:** 2025-05-21

**Authors:** Bai Li, Xin Yang, Sayed M. Ismail, Asma Gheisari

**Affiliations:** 1 School of Foreign Languages, China Pharmaceutical University, Nanjing City, Jiangsu Province 211198 China; 2 Zhuhai Sports School, Zhuhai City, Guangdong 519000 China; 3 Department of English Language, College of Sciences and Humanities, Prince Sattam bin Abdulaziz University, Al-kharj, Saudi Arabia; 4 Payame Nour University of Ahvaz, Ahvaz, Iran

**Retraction Note: BMC Psychology (2024) 12:19**

10.1186/s40359-023-01511-w

The editor and the publisher have retracted this article. The article was submitted to be part of a guest-edited issue. An investigation by the publisher found a number of articles, including this one, with a number of concerns, including but not limited to compromised editorial handling and peer review process, inappropriate or irrelevant references or not being in scope of the journal or guest-edited issue. Based on the investigation’s findings the editor therefore no longer has confidence in the results and conclusions of this article.

Authors Asma Gheisari and Sayed M. Ismail disagree with this retraction. The rest of the authors have not responded to correspondence regarding this retraction.

